# Application of Factorial and Doehlert Designs for the Optimization of the Simultaneous Separation and Determination of Antimigraine Drugs in Pharmaceutical Formulations by RP-HPLC-UV

**DOI:** 10.1155/2019/9685750

**Published:** 2019-08-15

**Authors:** Sami Jebali, Chaouki Belgacem, Mohamed Radhouen Louhaichi, Senda Bahri, Latifa Latrous El Atarche

**Affiliations:** ^1^Laboratoire National de Contrôle des Médicaments, 11 Bis Rue Jebel Lakhdar Bab Saadoun, 1006 Tunis, Tunisia; ^2^Université de Tunis El Manar, Faculté des Sciences de Tunis, Laboratoire de Chimie Analytique et Electrochimie, Campus Universitaire El Manar II 2092, Tunis, Tunisia; ^3^Institut National de Recherche et d' Analyse Physico-Chimique, Technopole, 2020 Sidi Thabet, Ariana, Tunis, Tunisia; ^4^Université de Tunis El Manar, Institut Préparatoire Aux Etudes d'Ingénieurs d'El Manar, B.P.244 El Manar II 2092, Tunis, Tunisia

## Abstract

A sensitive, precise, accurate, and specific isocratic reversed-phase high-performance liquid chromatographic (RP-HPLC) method for the simultaneous separation and determination of zolmitriptan, naratriptan, dihydroergotamine, ketotifen, and pizotifen in pharmaceutical formulations has been developed and validated. An experimental design was applied for the optimization of the chromatographic parameters. A two-level full factorial 2^*k*^ was used for studying the interaction between the variables to be optimized: the percentage of acetonitrile in the mobile phase, mobile-phase pH, nature of the buffer, and column oven temperature. The most significant parameters are the percentage of acetonitrile and the mobile-phase pH. These significant parameters were optimized using the Doehlert matrix. The optimum separation was achieved by means of a Waters XBridge C18 column (250 mm × 4.6 mm, 5 *μ*m) with a mobile phase consisting of acetonitrile and a 10 mM sodium perchlorate buffer (38 : 62, v/v) at a flow rate of 1.0 mL·min^−1^ and UV detection at 220 nm. The selectivity, method linearity, accuracy, and precision were examined as part of the method validation. The described method shows excellent linearity over a range of 30 to 70 *μ*g·mL^−1^ for all compounds with correlation coefficients higher than 0.995. The standard deviations of the intraday and interday precision were between 0.75 and 1.94%. The validated method was successfully applied to perform routine analysis of these compounds in different pharmaceutical products such as syrups and tablets. In the presence of some preservatives, it was found that there were no peaks at the related peak locations.

## 1. Introduction

Migraine is the third most prevalent and sixth most disabling medical illness in the world. It consists of headache attacks lasting 4–72 h, of moderate to severe intensity, associated with nausea and photo- and phonophobia [1, 2]. This pathology affects 10% of the global adult population between the age of 25 and 55 with highest prevalence in women [3, 4]. Antimigraine therapy includes currently potent serotonin 5-HT13/1D receptor agonists, a collectively known triptan drug class [5, 6]. They work by stimulating serotonin receptors in the brain. This is the reason why the role of 5-hydroxytryptamine (5-HT) as the central mediator of migraine attack has received much attention [7–12].

Zolmitriptan (Zol), naratriptan (Nara), dihydroergotamine (DHE), and pizotifen malate (Pizo) are recommended as first-line drugs for acute migraine treatment. Clinical studies have shown Zol to be effective and well tolerated [13]. It stimulates serotonin receptors in the brain causing blood vessels to narrow down, and consequently, it relieves the pain of migraines [14]. Likewise, Nara, a selective serotonin agonist, acts on 5-HT1 receptors to cause the vasoconstriction of cranial arteries. It is used also for the acute treatment of the headache phase of migraine attacks [15]. DHE, a semisynthetic ergopeptide, is widely used to prevent or treat vascular headaches such as migraine and cluster headaches [16, 17]. It acts by the stimulation of serotonergic receptors of neurons of the capacitance vessels [18, 19]. In addition, Pizo is an H1 receptor antagonist used for the prophylaxis of migraine [20, 21].

Various analytical procedures have been implemented for the determination of these compounds in pharmaceutical formulations and plasma samples. High-performance liquid chromatography (HPLC) with ultraviolet-visible detection [22–24], HPLC with fluorescence detection [16, 17, 25, 26], HPLC with coulometric detection [27, 28], and HPLC with mass spectroscopy [29–35] are the most used techniques. Additionally, a micellar electrokinetic capillary chromatography has been developed and validated to allow the analysis of Nara in pharmaceutical products [36]. The quantification of Pizo is also determined by various atomic absorption methods, colorimetric methods, and potentiometric methods [28, 37]. The electrochemical behavior of Nara by developing a differential pulse voltammetric assay was examined to study its content uniformity in the tablet form [38]. Recently, a new method for selective determination of triptans in rat plasma has been developed with an efficient zirconia-based reversed-phase chromatography [39]. Screen-printed silver electrodes are shown as cost-effective surface-enhanced Raman scattering substrates for the sensitive and quantitative detection of naratriptan [15].

Most of the optimized separation methods cited in the literature for the analysis of antimigraine drugs by RP-HPLC involve a variation of a large number of variables in the separation process. To our knowledge, no paper carried out changes of experimental conditions all along the chromatographic run, for the separation of antimigraine drugs. For this reason, it is more effective and time-saving to resort to the experimental design procedure.

Being a laboratory having as the main task the quality control of drugs intended for use in the local market, it was of big interest for our team to develop a reliable analytical technique for the detection and quantification of Zol, Nara, DHE, Keto, and Pizo in various pharmaceutical products. So far, to the best of our knowledge, no reversed-phase high-performance liquid chromatographic techniques with ultraviolet-visible detection (RP-HPLC-UV) were reported in the literature for the simultaneous separation and quantification of triptan pharmaceutical formulations. In the first step of this work, a two-level full factorial design was used to study the effects of factors which influence and interfere in the simultaneous separation and determination of Zol, Nara, DHE, and Pizo in both raw materials and pharmaceutical formulations. In the second step, the most influential factors are optimized using the response surface methodology with a Doehlert design. This method was fully validated according to the International Conference on Harmonization (ICH) validations rules [40]. The applicability of the developed method was fully and successfully verified by the analysis of several commercialized pharmaceutical products from the local market.

## 2. Materials and Methods

### 2.1. Reagents and Chemicals

**Figure 1:**
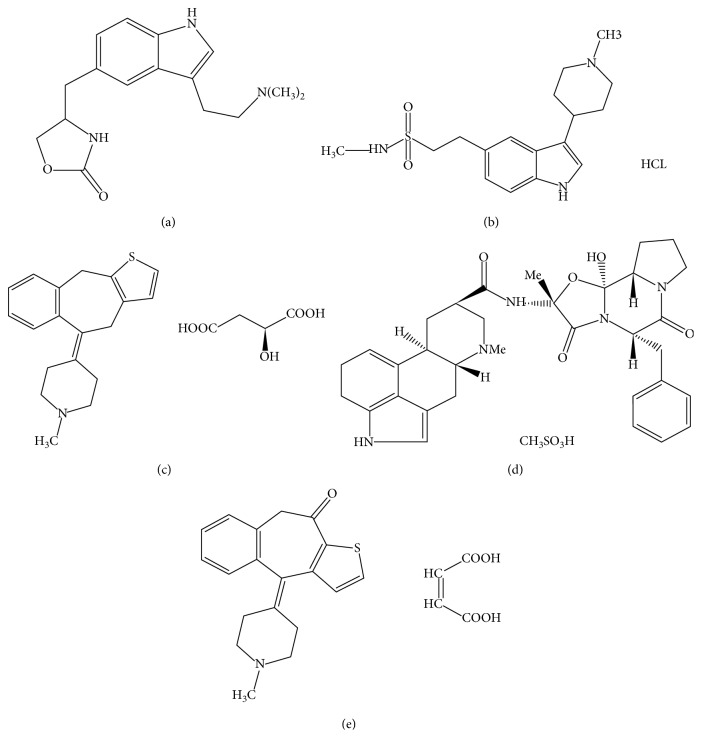
Chemical structures of studied compounds. (a) Naratriptan (Nara). (b) Zolmitriptan (Zol). (c) Pizotifen malate (Pizo). (d) Dihydroergotamine mesilate (DHE). (e) Ketotifen fumarate (Keto).

The reference standards of Pizo, DHE, Nara, Keto, and Zol with a purity of 99.99% were obtained from Sigma-Aldrich (France). The chemical structures of all studied compounds are shown in Figure 1. HPLC-grade methanol (MeOH) and acetonitrile (ACN) were purchased from Prolabo (Paris, France). High-purity water was prepared by using the Diamond Reverse Osmosis System (United Kingdom). Sodium perchlorate, perchloric acid, sodium acetate, acetic acid, orthophosphoric acid, and sodium hydroxide were purchased from Prolabo (Paris, France). Sodium dihydrogenphosphate was purchased from Carlo Erba (France).

The commercialized pharmaceutical products used were Zomig tablets (2.5 mg Zol) produced by AstraZeneca United Kingdom; Naramig tablets (2.5 mg Nara) marketed by GlaxoSmithKline, France; Ikaran LP tablets (5 mg DHE) manufactured by Pierre Fabre Medicament Production in Tunisia; Pizofen syrup (0.25 mg/5 mL Pizo) manufactured by Simed, Tunisia; and Tefanyl tablets (1 mg Keto) and Pizofen tablets (0.5 mg Pizo) manufactured by Saiph, Tunisia.

### 2.2. Chromatographic Conditions

LC analyses were performed on an Agilent model 1200 consisting of a quaternary pump, an autosampler, a vacuum degasser, and a thermostated column compartment. Separation was achieved on a Waters XBridge® ODS column (250 mm × 4.6 mm ID, 5 *μ*m particle size).

The mobile phase was composed of 38 : 62 (v/v) acetonitrile/10 mM sodium perchlorate buffer (pH 3.5). The system was equilibrated with the mobile phase before injection, and the flow rate was 1.0 mL·min^−1^. The detector consisted of a diode-array detector (DAD) UV-visible model 1200.

### 2.3. Preparation of Solutions

Stock solutions of 500 *μ*g·mL^−1^ of each compound were prepared in MeOH. These solutions were used to prepare the working solutions by serial dilutions in ultrapure water in the range 30–70 *μ*g·mL^−1^.

Oral suspension samples and syrup were diluted with ultrapure water at a concentration of 50 *μ*g·mL^−1^. The obtained solutions were sonicated for 10 min and were filtered with a 0.45 *μ*m filter prior to injection into the HPLC system.

Twenty units of tablet and capsule samples were accurately weighted and powdered separately in a mortar. An equivalent weight of 5 mg of the studied compound was dissolved in 100 mL ultrapure water. After 15 min of mechanical shaking, the solution was filtered through a 0.45 *μ*m Millipore filter. All preparations were performed in three replicates.

## 3. Results and Discussion

The nature of the stationary phase was chosen on the basis of previous works [4, 32, 41]. The majority of the published HPLC method uses octadecylsilica columns (C18) for the separation of antimigraine drugs. For the selection of the wavelength, as shown in the PDA spectra, the studied compounds have a good absorbance at 220 nm.

### 3.1. Study of the Influence of Factors on Resolution and Run Time

In order to quantify the influence of operating parameters on the simultaneous separation of antimigraine drugs, four main factors were chosen: % of ACN in the mobile phase (*U*_1_), mobile-phase pH (*U*_2_), nature of the buffer (*U*_3_), and column oven temperature (*U*_4_). These variables with their respective domain are chosen on the basis of the previous work [41] and preliminary studies, and they are presented in Table 1. Experiments were performed on a two-level full factorial design 2^*k*^ [42, 43]. In these types of designs, variables (*k*) are set at two levels (minimum and maximum) normalized as −1 and +1. The experimental response (*Y*) associated with a 2^*k*^ factorial design (for four variables) is represented by a linear polynomial model with interaction as follows:

(1) $$\begin{array}{c} Y=b_{0}+b_{1}X_{1}+b_{2}X_{2}+b_{3}X_{3}+b_{4}X_{4}+b_{12}X_{1}X_{2}+b_{13}X_{1}X_{3}+b_{23}X_{2}X_{3}+b_{14}X_{1}X_{4}+b_{24}X_{2}X_{4}+b_{34}X_{3}X_{4}+b_{123}X_{1}X_{2}X_{3}+b_{124}X_{1}X_{2}X_{4}+b_{134}X_{1}X_{3}X_{4}+b_{234}X_{2}X_{3}X_{4}+b_{1234}X_{1}X_{2}X_{3}X_{4}, \end{array}$$

where *Y* is the experimental response, *X*_*i*_ the coded variable (−1 or +1), *b*_*i*_ the estimation of the principal effect of the factor *i* for the response *Y*, and *b*_*ij*_ the estimation of the interaction effect between factors *i* and *j* for the response *Y*. The coefficients of the equation model were calculated in the experimental field, as listed in Table 1.

The experimental design and results are presented in Table 2. The responses of interest are resolution *R*_s_ between Zol and Nara (*Y*_1_) and run time (*Y*_2_).

According to the obtained results, the coefficients of the polynomial model were calculated using the NEMROD software [44]:

(2) $$\begin{array}{c} Y_{1}=3.54-1.33X_{1}+0.13X_{2}+1.31X_{3}-0.12X_{4}+0.01X_{1}X_{2}+0.28X_{1}X_{3}-0.01X_{2}X_{3}-0.02X_{1}X_{4}-0.01X_{2}X_{4}-0.06X_{3}X_{4}-0.07X_{1}X_{2}X_{3}-0.01X_{1}X_{2}X_{4}-0.01X_{1}X_{3}X_{4}+0.01X_{2}X_{3}X_{4}+0.01X_{1}X_{2}X_{3}X_{4},\\ Y_{2}=34.688-17.688X_{1}+11.313X_{2}-0.125X_{3}-0.6X_{4}-5.563X_{1}X_{2}+7.0X_{1}X_{3}-0.375X_{2}X_{3}-0.025X_{1}X_{4}-0.275X_{2}X_{4}-0.337X_{3}X_{4}+2.75X_{1}X_{2}X_{3}-0.15X_{1}X_{2}X_{4}-0.213X_{1}X_{3}X_{4}-0.287X_{2}X_{3}X_{4}-0.162X_{1}X_{2}X_{3}X_{4}. \end{array}$$

**Figure 2:**
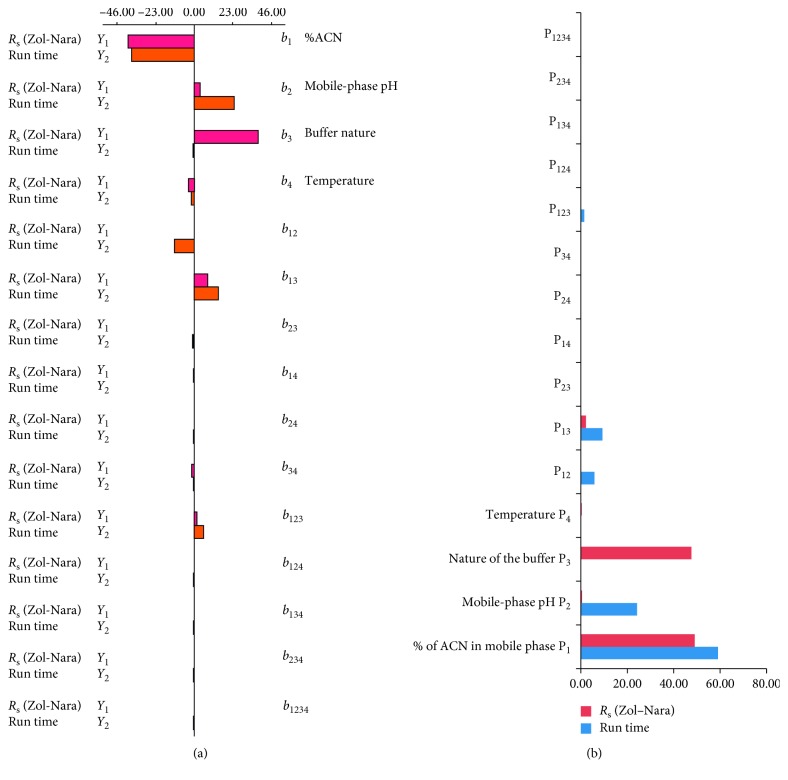
(a) Graphic analysis of effects. (b) Pareto graphic analysis.

The effects and interactions of various investigated factors are presented in Figure 2(a). This figure shows that the most influential factors are % ACN, mobile-phase pH, and nature of the buffer. However, the temperature and the interaction variables have negligible effects. The % ACN has a negative effect on the studied response. As expected, the variation of solute retention with the mobile-phase composition follows the usual trend and shows a faster elution of all studied compounds upon increasing ACN percentage. The observed effect is due to a decrease in the dielectric constant of the mixture after progressively increasing the ratio of the organic modifier and, consequently, leads to a decrease in the retention factor. Mobile-phase pH is the second most significant factor of the separation of triptan drugs. Its effect is positive, especially on time analysis. The changes in the retention as a function of the pH result from the changes in the ionization form of these solutes, which is pKa dependent. The elution of Zol, Nara, and Keto was rapid compared to that of DHE and Pizo. Their retention times remained almost unchanged over the studied pH range because of their high pKa values (the pKa of Zol, Nara, and Keto was, respectively, 9.64, 9.7, and 8.43). In fact, at this pH range, Zol, Nara, and Keto were protonated, so they were not retained by the reversed phase. However, the retention time of DHE and Pizo increased with the pH because of their lower pKa values (6.75 and 6.95, respectively). Sodium dihydrogenphosphate and sodium perchlorate buffers (pH = 3.5) were tested to investigate their effect on both peak shape and retention time. Their proportions in the mobile phase were adjusted in order to obtain an acceptable separation in comparable analysis time. It appears that the retention order of the 5 compounds with the 2 buffers was similar. However, the retention time of the 5 compounds was affected by the nature of the solution buffer and especially by the perchlorate buffer. Pareto analysis [45] gives more significant information to interpret these results. In fact, this analysis calculates the percentage effect of each factor on the response, according to the following relation:

(3) $$\begin{array}{c} P_{i}=\left(\frac{b_{i}^{2}}{\sum b_{i}^{2}}\right)\times 100,\;i\neq 0. \end{array}$$

Figure 2(b) presents the Pareto graphic analysis. The results show that the % of acetonitrile in the mobile phase and mobile-phase pH are the most determining factors of the (separationary) separation of the studied compounds. Therefore, 83.30% of the response is brought about by these two factors. The % of acetonitrile in the mobile phase represents 59.12% of the response.

### 3.2. Optimization of Chromatographic Conditions

In the second step, to determine optimum factors that ensure the chromatographic separation of the target antimigraine drugs in pharmaceutical formulations, predominant factors such as mobile-phase composition and mobile-phase pH were investigated.

For this study, the response surface methodology based on empirical mathematical modeling was used. A second-order polynomial model was used to study the possible nonlinear effects and curvature in the field of study:

(4) $$\begin{array}{c} Y=b_{0}+b_{1}X_{1}+b_{2}X_{2}+b_{11}X_{1}^{2}+b_{22}X_{2}^{2}+b_{12}X_{1}X_{2}. \end{array}$$

A Doehlert uniform shell design is performed [46–48]. The factors are given in the form of coded variables (*X*_*i*_) without units in order to permit comparison of factors of different natures. The transformation of natural variables (*U*_*i*_) into coded corresponding variables (*X*_*i*_) is made on the basis of the following equation:

(5) $$\begin{array}{c} X_{i}=\left[\frac{U_{i}-\bar{U_{i}}}{\Delta U_{i}}\right]\alpha , \end{array}$$

where *X*_*i*_ is the value taken by the coded variable *i*; *U*_*i*_ is the value taken by the factor *i*; $\bar{U_{i}}$ is the value taken by the factor *i* in the centre of the experimental field; Δ*U*_*i*_ is the range of variation of the factor *I*; and *α* is the maximum coded value of *X*_*i*_: *X*_1_ = 1, *X*_2_ = 0.866, and *X*_3_ = 0.816. The levels of the independent variables (effective variables *U*_*i*_) were calculated according to these following relations:

(6) $$\begin{array}{c} U_{1}=7.5X_{1}+32.5,\\ U_{2}=2.9X_{2}+4.5. \end{array}$$

Replicates at the central level of the variables were performed in order to validate the model by means of an estimation of experimental variance. The experiment at the centre (experiment number 7) was carried out three times (Table 3) in order to obtain an estimation of the experimental error. According to these obtained results, the coefficients of the polynomial model were calculated using the NEMROD software [44].

**Figure 3:**
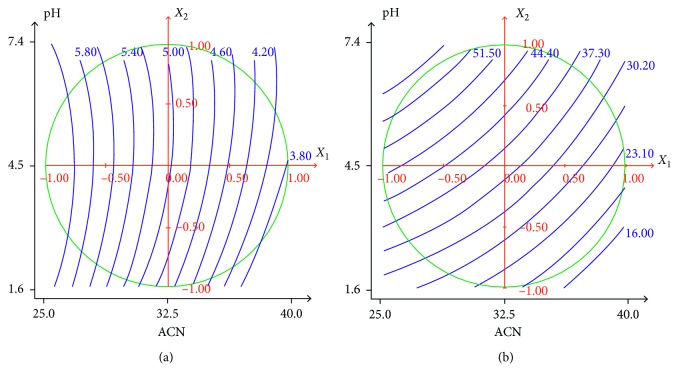
Variation of (a) *R*_s_ (*Y*_1_) and (b) run time (*Y*_2_) as a function of % ACN and pH.

Figure 3 shows typical response surface profiles drawn versus the main factors: % of acetonitrile and mobile-phase pH, using the NEMROD software [44].

The analysis of the isoresponse curves at the chosen experimental field delimited by a circle shows that the compromise between the maximum of resolution and less run time was obtained where the percentage of acetonitrile and mobile-phase pH range, respectively, between 35 and 40% and 3 and 4. To determine an acceptable compromise zone, responses were simultaneously optimized by using the desirability function approach, included in the NEMROD software. Consequently, the optimal working conditions were obtained when studied antimigraine products were eluted with 38% acetonitrile and 62% perchlorate buffer (10 mM) with pH 3.5.

**Figure 4:**
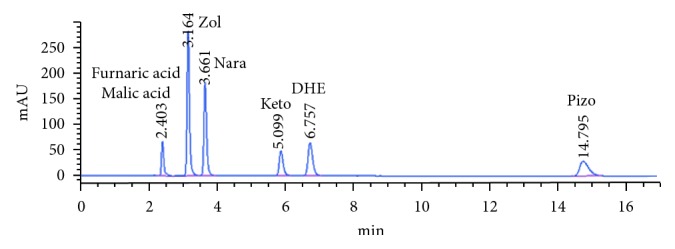
Representative chromatogram of standard active compounds. Mobile phase: ACN-sodium perchlorate buffer (10 mM) (38 : 62, v/v) at pH 3.5; column: Waters XBridge C18 (250 mm × 4.6 mm, 5 *μ*m); detection at 220 nm; flow rate: 1 mL·min^−1^.

As can be seen from Figure 4, the optimal conditions for the separation of the studied compounds by RP-HPLC provide satisfactory resolution with a short time analysis.

### 3.3. Validation of the Method

The optimized method was validated in relation to the following properties: specificity, linearity, precision (repeatability and intermediate precision), accuracy, recovery, limit of detection, and limit of quantification. Recoveries were determined at five different concentration levels, and linearity, accuracy, and precision were determined within and between three different days.

#### 3.3.1. Specificity

**Figure 5:**
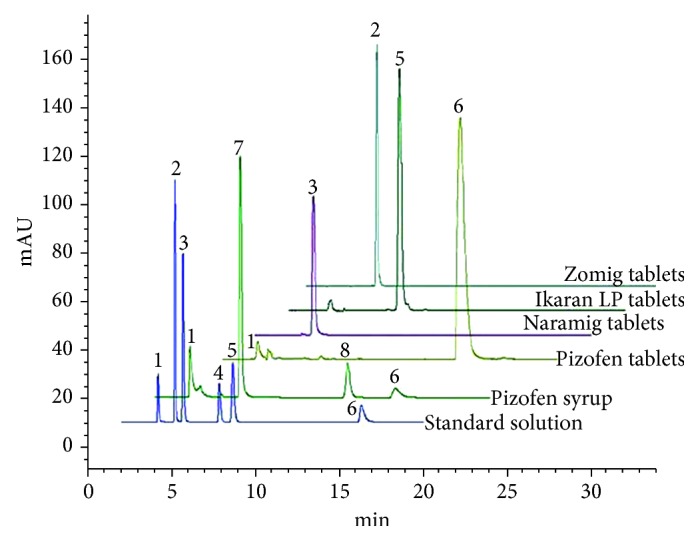
Typical chromatograms of real samples: Zomig tablets, Naramig tablets, Ikaran LP tablets, Pizofen tablets, and Pizofen syrup. Mobile phase: 38 : 62 (v/v) ACN : sodium perchlorate buffer (10 mM) at pH 3.5; column: Waters XBridge C18 (250 mm × 4.6 mm, 5 *μ*m); detection at 220 nm; flow rate: 1.0 mL·min^−1^. Identification: 1 = maleic acid, 2 = zolmitriptan, 3 = naratriptan, 4 = ketotifen, 5 = dihydroergotamine, 6 = pizotifen, 7 = methylparaben, and 8 = propylparaben.

According to the International Conference on Harmonization (ICH) validations rules (Validation of Analytical Procedures: Text and Methodology Q2 (R1)), specificity is the ability to assess unequivocally the target compound in the presence of components, which may be expected to be present [40]. The resolution factors between all peaks in standard solutions (Figure 4) and in real samples (Figure 5) are satisfactory, reflecting the specificity of the method. As shown in Figure 5, the presence of preservative peaks did not interfere with the studied compounds. Peaks of the placebo components were adequately separated from those of active compounds which indicates the selectivity of the method.

On the contrary, as shown in Table 4, the purity factors of all peaks are more than 950, and the peak purity of each active compound and PDA spectra shows that no interferences with any component (related impurities, degradation products, or excipients) are observed. Therefore, the proposed method is applicable to the selective determination of these compounds.

#### 3.3.2. Linearity

The linearity of the method was evaluated by injecting solutions of each antimigraine drug over a wide concentration range (30 to 70 *μ*g·mL^−1^). For each analyte, calibration curves were constructed. Calibration data could fit a linear model for all analytes with typical correlation coefficients exceeding 0.995, as shown in Table 5.

*F* values (Fisher's test for the existence of a significant slope) that are greater than the Fisher *F* critical value deduced from the table (*α*, 1, and *N* − 2) for *α* = 0.05 and *N* = 15 are 4.67 for all the analytes, since we can conclude that the regression is acceptable at the risk of error of 5%.

#### 3.3.3. Precision

Method precision was determined by replicate analyses of 6 independent preparations at 100% of the test concentration according to ICH validation rules [40]. The intermediate precision was obtained by repeating the intra-assay experiment for three days.

The repeatability and intermediate precision values were calculated as relative standard deviation (RSD) of the found values. The mean RSD values for intra- and interday precision varied from 0.78 to 1.84% and from 0.75 to 1.94%, respectively (Table 6).

#### 3.3.4. Accuracy and Recovery

The accuracy of the method was evaluated in triplicate by recovery tests at five concentration levels (30, 40, 50, 60, and 70 *μ*g·mL^−1^). Therefore, recovery tests were performed by spiking different samples at five levels, and the results obtained as well as the respective relative standard deviation (RSD) are presented in Table 7. The higher values of recoveries and the lower values of the RSD of the assay indicate that the method is precise and accurate. The results depict that the present method is useful for bulk drug analysis as well as commercial pharmaceuticals in different forms and different types of formulations.

#### 3.3.5. Limit of Detection (LOD) and Limit of Quantification (LOQ)

The LOD and LOQ were estimated for all compounds based on signal-to-noise ratio (S/N). The baseline noise was measured in a blank experiment in the region of retention time of the compound using chromatographic software. The limits of detection (S/N = 3) and of quantification (S/N = 10) of Pizo, Keto, DHE, Nara, and Zol ranged from 0.075 to 0.10 *μ*g·mL^−1^ and 0.25 to 0.33 *μ*g·mL^−1^, respectively.

### 3.4. Application of the Method

The potential of the method for the analysis of pharmaceutical formulations from the local market such as tablets and syrup has been demonstrated. The outcome of this study has shown a good agreement between the experimental and label claims (Table 8). The recovery percentage, with respect to the label claims, ranged between 97.45 and 102.60%, indicating a good accuracy of the proposed method. In order to further assess the accuracy of the proposed method and taking into consideration the matrix effect, a new set of recovery experiments were carried out by spiking the drug solutions with known amounts of standard target compound solutions.

The typical chromatograms related to the separation of the five end products are illustrated in Figure 5. The peaks were identified by comparison of the retention time of the separated compounds and standards. No interference between peaks was observed in the chromatograms of the commercial formulations under the described conditions. Therefore, the excipients present in the commercial preparations have no interference with the analysis of Zol, Nara, Keto, DHE, and Pizo.

## 4. Conclusion

Applying fractional factorial design (FFD) with the purpose of screening of variables and Doehlert design for optimization of the screened variables, the simultaneous determination of Zol, Nara, DHE, and Pizo in pharmaceutical preparations was conducted by reversed-phase high-performance liquid chromatography (RP-HPLC).

The factorial designs have demonstrated that the percentage of acetonitrile in the mobile phase and mobile-phase pH are the most influent parameters of optimization of chromatographic separation. In addition, the method was validated according to ICH guidelines; the results indicate that the method is sensitive, precise, accurate, and applicable to various commercial pharmaceutical preparations: syrups and tablets containing Zol, Nara, DHE, and Pizo. Therefore, the method can be used for routine quality control analysis in the pharmaceutical environment. The results of this study demonstrate the benefit of applying the experimental design methodology in selecting optimum conditions for the determination of drugs in pharmaceutical formulations.

## Figures and Tables

**Table 1:** Investigated variables and their levels studied in the 2^4^ factorial design.

*U*_*i*_	Factor	Value	
		−1	+1
*U*_1_	% ACN	25	40
*U*_2_	Mobile-phase pH	2	7
*U*_3_	Buffer nature	Phosphate	Perchlorate
*U*_4_	Temperature	30	40

**Table 2:** Factorial design, experimental conditions, and experimental results.

Experiment no.	Experimental design					Experimental plan		Results		
	*X*_1_	*X*_2_	*X*_3_	*X*_4_	*U*_1_	*U*_2_	*U*_3_	*U*_4_	*Y*_1_	*Y*_2_ (min)
1	−1	−1	−1	−1	25	2	Phosphate	30	3.7	40
2	1	−1	−1	−1	40	2	Phosphate	30	0.6	6.8
3	−1	1	−1	−1	25	7	Phosphate	30	4.1	80
4	1	1	−1	−1	40	7	Phosphate	30	0.8	13.5
5	−1	−1	1	−1	25	2	Perchlorate	30	6.0	32
6	1	−1	1	−1	40	2	Perchlorate	30	3.8	16
7	−1	1	1	−1	25	7	Perchlorate	30	6.1	60
8	1	1	1	−1	40	7	Perchlorate	30	4.2	34
9	−1	−1	−1	1	25	2	Phosphate	40	3.6	39
10	1	−1	−1	1	40	2	Phosphate	40	0.5	6.7
11	−1	1	−1	1	25	7	Phosphate	40	4.0	79
12	1	1	−1	1	40	7	Phosphate	40	0.6	13.5
13	−1	−1	1	1	25	2	Perchlorate	40	5.7	31
14	1	−1	1	1	40	2	Perchlorate	40	3.4	15.5
15	−1	1	1	1	25	7	Perchlorate	40	5.8	58
16	1	1	1	1	40	7	Perchlorate	40	3.8	30

*Y*_1_: resolution *R*_s_ (Zol-Nara); *Y*_2_: run time.

**Table 3:** Doehlert's experimental design, experimental conditions, and responses.

Experiment no.	Design of experiments		Operating conditions		Experimental conditions	
	*X*_1_	*X*_2_	ACN (%)	pH	*Y*_1_	*Y*_2_ (min)
1	1.0000	0.0000	40.0	4.5	4.01	23.00
2	−1.0000	0.0000	25.0	4.5	6.05	44.00
3	0.5000	0.8660	36.3	7.0	4.20	37.00
4	−0.5000	−0.8660	28.8	2.0	5.70	29.00
5	0.5000	−0.8660	36.3	2.0	3.80	16.00
6	−0.5000	0.8660	28.8	7.0	5.80	55.00
7	0.0000	0.0000	32.5	4.5	5.04	35.50
8	0.0000	0.0000	32.5	4.5	5.01	35.10
9	0.0000	0.0000	32.5	4.5	5.06	35.40

*Y*_1_: resolution *R*_s_ (Zol-Nara); *Y*_2_: run time.

**Table 4:** Purity factors of studied compounds.

Compound	Purity factor
Zol	991.315
Nara	999.962
Keto	984.863
DHE	980.589
Pizo	994.632

**Table 5:** Linearity details of selected compounds.

Compound	Equation	Correlation coefficient	*F* (Fisher)
Zol	*y*=64153.333*x*+16393.667	0.998	2535.39
Nara	*y*=51618.667*x* − 7266.333	0.997	2057.85
Keto	*y*=22653.000 *x* − 6153.333	0.997	2154.05
DHE	*y*=33653.000 *x* − 961.667	0.997	2121.47
Pizo	*y*=31270.333 *x* − 37732.333	0.996	2325.35

**Table 6:** Data on intra- and interday method precision.

Compound	Interday mean RSD (%)	Intraday mean RSD (%)
Zol	0.78	0.75
Nara	0.85	0.86
Keto	1.84	1.03
DHE	1.83	1.94
Pizo	1.41	1.63

**Table 7:** Method precision and accuracy for the recovery of studied compounds.

Compound	Amount spiked (*μ*g·mL^−1^)	Amount recovered (*μ*g·mL^−1^)	Mean recovery (%)	RSD (%)
*At 60% level*				
Zol	30	29.96	97.92	0.43
Nara	30	30.19	100.56	0.93
Keto	30	29.40	97.99	0.11
DHE	30	30.22	100.73	0.69
Pizo	30	30.58	100.94	0.16

*At 80% level*				
Zol	40	41.07	101.17	0.85
Nara	40	39.84	99.60	1.16
Keto	40	40.55	101.38	0.94
DHE	40	40.12	100.31	0.89
Pizo	40	40.28	99.95	1.54

*At 100% level*				
Zol	50	51.39	101.57	0.83
Nara	50	50.59	101.18	0.96
Keto	50	50.89	101.78	1.55
DHE	50	49.88	99.77	2.36
Pizo	50	50.55	100.49	1.90

*At 120% level*				
Zol	60	60.11	99.20	0.69
Nara	60	58.63	97.72	2.02
Keto	60	58.98	98.30	1.00
DHE	60	58.99	98.31	1.47
Pizo	60	58.74	97.41	0.77

*At 140% level*				
Zol	70	70.44	99.78	0.24
Nara	70	70.77	101.11	1.42
Keto	70	70.18	100.26	0.35
DHE	70	70.79	101.12	0.50
Pizo	70	71.35	101.50	0.57

**Table 8:** Content of industrial pharmaceutical forms with respect to the label amount claimed.

Sample	Compounds	Label content	Norms (%)	Found	Recovery (%)
Zomig tablets	Zol	2.5 mg/tablet	100.0 ± 5.0	2.44 mg/tablet	97.45
Naramig tablets	Nara	2.5 mg/tablet	100.0 ± 5.0	2.49 mg/tablet	99.87
Ikaran LP tablets	DHE	5 mg/tablet	100.0 ± 5.0	5.13 mg/tablet	102.60
Pizofen tablets Pizofen syrup	Pizo	0.5 mg/tablet 0.25 mg/5 mL	100.0 ± 5.0 100.0 ± 5.0	0.49 mg/tablet 0.24 mg/5 mL	98.04 98.24
Tefanyl tablets	Keto	1.0 mg/tablet	100.0 ± 5.0	0.99 mg/tablet	98.61

## Data Availability

The data used to support the findings of this study are available from the corresponding author upon request.

## Supplementary Materials

Supplementary Materials Figure S1: purity spectra of studied compounds. Figure S2: PDA spectra of antimigraine drugs. Figure S3: linearity graphs of selected compounds. Figure S4: placebo chromatograms of studied commercialized pharmaceutical products. Table S1: placebo compositions of studied commercialized pharmaceutical products.
