# Supplementary material for: Application of Factorial and Doehlert Designs for the Optimization of the Simultaneous Separation and Determination of Antimigraine Drugs in Pharmaceutical Formulations by RP-HPLC-UV
Source: Int J Anal Chem. 2019 Aug 15;2019:9685750. doi: 10.1155/2019/9685750 (PMC6714324; doi:10.1155/2019/9685750)
Supplement: Supplementary Materials — Figure S1: purity spectra of studied compounds. Figure S2: PDA spectra of antimigraine drugs. Figure S3: linearity graphs of selected compounds. Figure S4: placebo chromatograms of studied commercialized pharmaceutical products. Table S1: placebo compositions of studied commercialized pharmaceutical products. [file 9685750.f1.docx]

**Application of factorial and Doehlert designs for the optimization of the simultaneous separation and determination of antimigraine drugs in pharmaceutical formulations by RP-HPLC-UV**

Sami Jebali^1,2^, Chaouki Belgacem^2^, Mohamed Radhouen Louhaichi^1^, Senda Bahri^1^, Latifa Latrous El Atarche^3,4*^

*^1^Laboratoire National de Contrôle des Médicaments, 11 bis Rue Jebel Lakhdar Bab Saadoun, 1006 Tunis, Tunisie.*

*^2^Institut National de Recherche et d' Analyse Physico-Chimique, Technopole, 2020, Sidi Thabet, Ariana-Tunis ,Tunisie.*

*^3^Université de Tunis El Manar, Faculté des Sciences de Tunis, Laboratoire de Chimie Analytique et Electrochimie, Campus universitaire El Manar II 2092, Tunis, Tunisie.*

*^4^Université de Tunis El Manar, Institut Préparatoire aux Etudes d’Ingénieurs d’El Manar, B.P.244 El Manar II 2092 Tunis, Tunisie****.***

**Supporting information**

**Fig. S1:** Purity spectra of studied compounds.

**Fig. S2:** PDA spectra of antimigraine drugs.

**Fig. S3:** linearity graphs of selected compounds.

**Fig.S4**: Placebo chromatograms of studied commercialized pharmaceutical products.


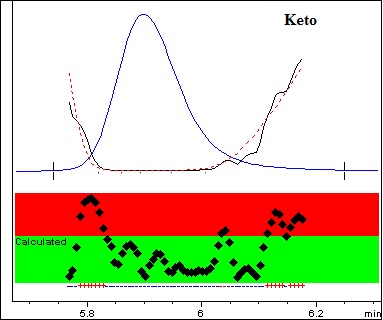

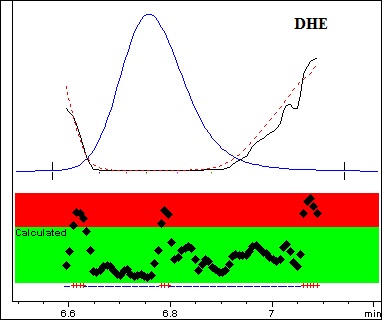


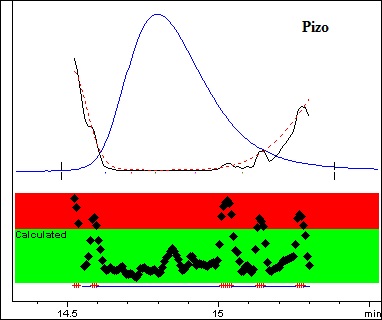

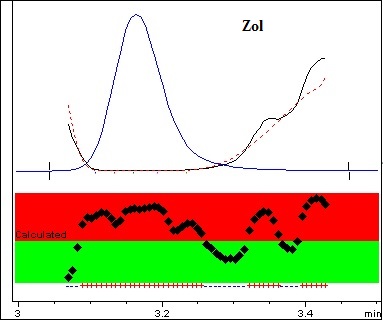


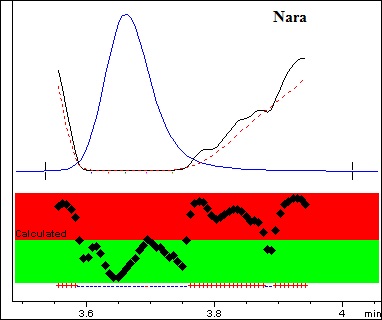


**Fig.S1**


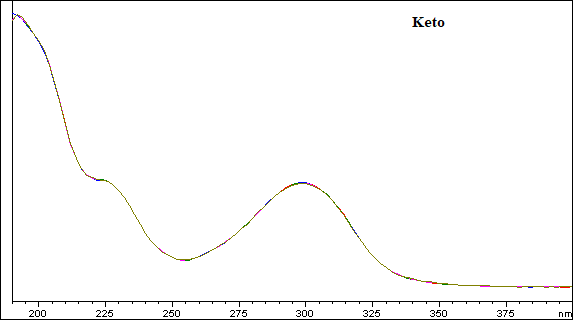

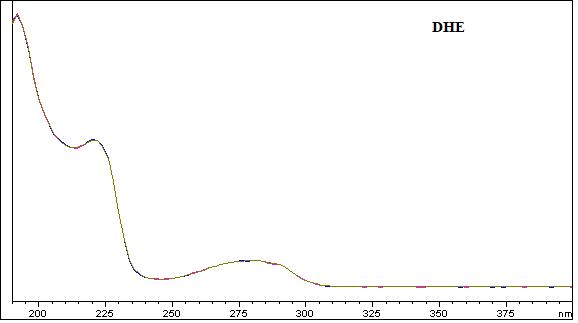


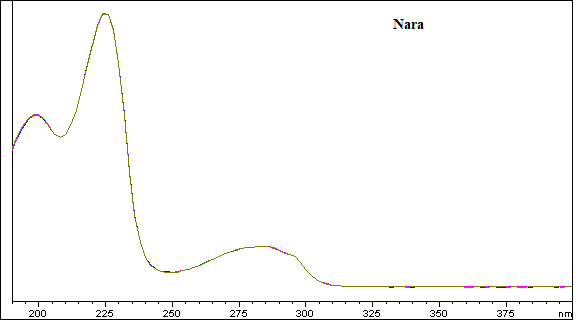

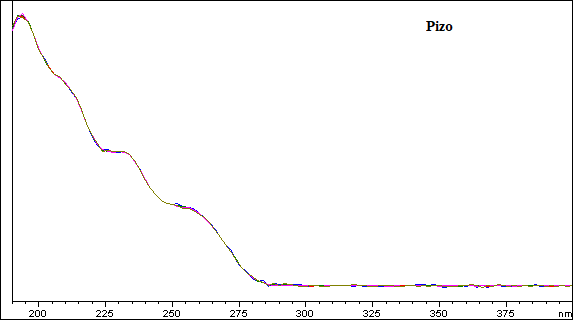


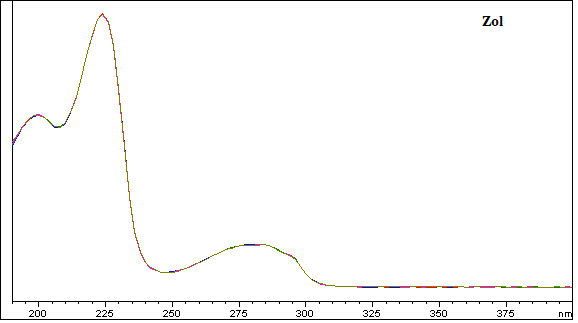


**Fig.S2**

**Pizo**

**DHE**

**Fig.S3**


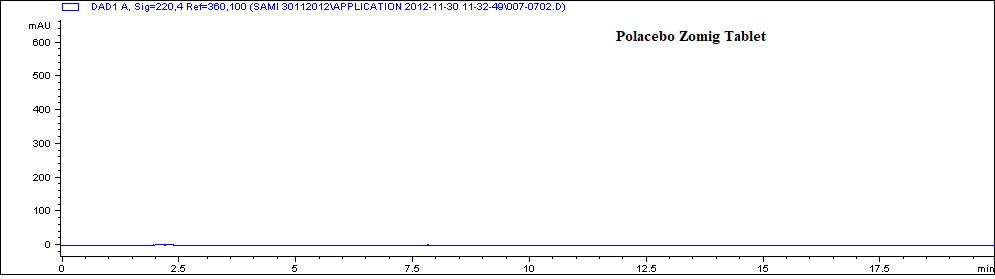


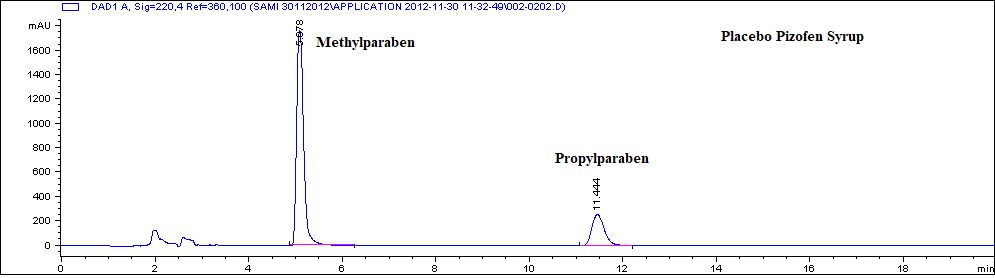


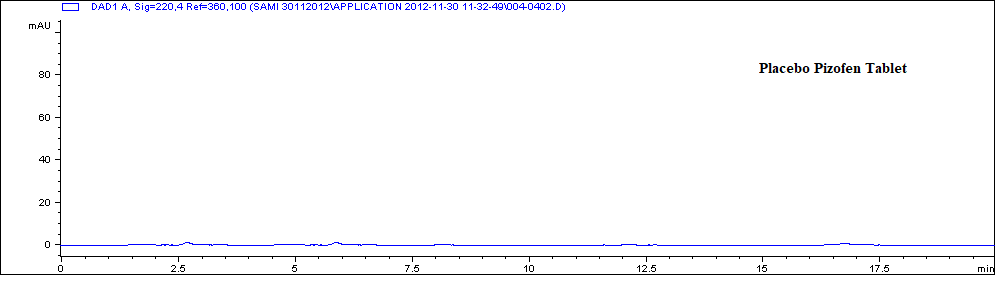


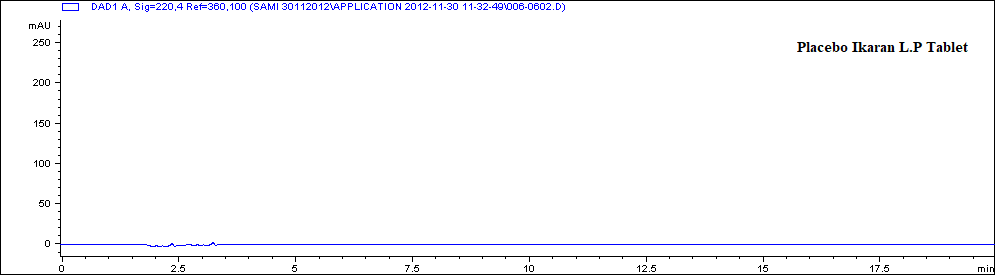


**
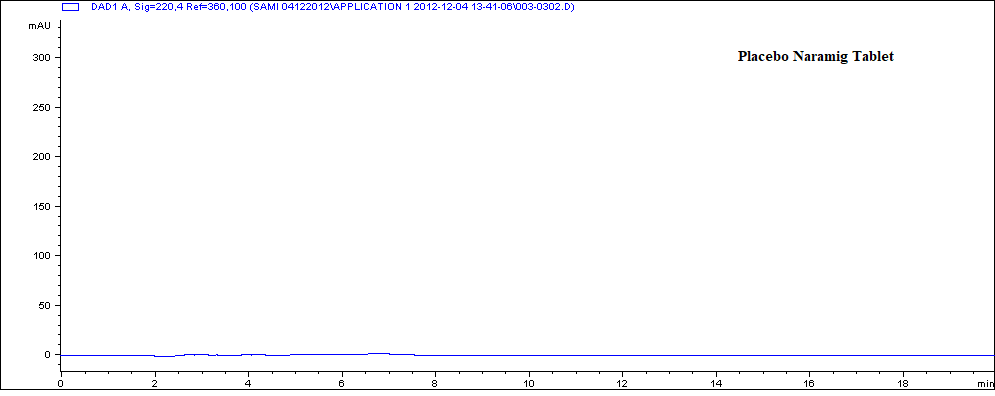
**

**Fig.S4**

**Table S1.** Placebo compositions of studied commercialized pharmaceutical products.

| **Pizotifen tablet** | **Zomig tablet** | **Naramig tablet** | **Ikaran LP tablet** | **Pizofen Sirop** |
| --- | --- | --- | --- | --- |
| - Povidone - Lactose monohydrate - Maize starch - Magnesium stearate - Microcrystalline cellulose | - Lactose anhydrous - Microcrystalline cellulose - Sodium starch glycolate - Magnesium stearate | - Lactose anhydrous - Microcrystalline cellulose - Sodium starch glycolate - Magnesium stearate | - Lactose - Hypromellose - Maize starch - Magnesium stearate - colloidal silica anhydrous | - Citric acid - anhydrous disodium - phosphate anhydrous - Methylparaben - Propylparaben - strawberry aroma - Sucrose - propylene glycol |
